# Unraveling the mechanisms of synapse formation and axon regeneration: the awesome power of *C. elegans* genetics

**DOI:** 10.1007/s11427-015-4962-9

**Published:** 2015-11-13

**Authors:** JIN YiShi

**Affiliations:** Howard Hughes Medical Institute, Section of Neurobiology, Division of Biological Sciences, University of California, San Diego CA 92093, USA

**Keywords:** presynaptic active zone, DLK kinase, microtubule dynamics, EFA-6, RPM-1, SYD-2, Liprin, ubiquitin E3 ligase, axon injury, laser axotomy

## Abstract

Since *Caenorhabditis elegans* was chosen as a model organism by Sydney Brenner in 1960’s, genetic studies in this organism have been instrumental in discovering the function of genes and in deciphering molecular signaling network. The small size of the organism and the simple nervous system enable the complete reconstruction of the first connectome. The stereotypic developmental program and the anatomical reproducibility of synaptic connections provide a blueprint to dissect the mechanisms underlying synapse formation. Recent technological innovation using laser surgery of single axons and *in vivo* imaging has also made *C. elegans* a new model for axon regeneration. Importantly, genes regulating synaptogenesis and axon regeneration are highly conserved in function across animal phyla. This mini-review will summarize the main approaches and the key findings in understanding the mechanisms underlying the development and maintenance of the nervous system. The impact of such findings underscores the awesome power of *C. elegans* genetics.

The Spanish neuroanatomist Santiago Ramon Y Cajal laid down the foundation for modern neuroscience by proposing the Neuron Doctrine, which describes information flow within the nervous system to be directional and contiguous through junctions between neurons. The English neurosurgeon Charles Sherrington introduced the term “synapse” to refer to such junctional structures. About 50 years later, using electron microscopy, two teams of scientists, Palay and Palade, and De Robertis and Bennett, provided the “seeing-is-believing” proof for a synapse to be an asymmetric subcellular structure, with a cluster of synaptic vesicles located at the presynaptic side, opposing to the postsynaptic partner through a visible gap across the junction [1]. How synapses are built and function has been a subject of investigation for decades, and remains to be at the center of attentions in multiple fields from electrophysiology to cell biology and molecular genetics.

Shortly after his work on elucidating the development of the nervous system, Ramon Y Cajal also made extensive and incisive observations on the degeneration and regeneration of the nervous system [2]. He noted the striking difference of axon regeneration in different environments of the nervous system. The periphery nerves can regenerate, ultimately regain full or partial function, whereas the nerves in the central nervous system sprout, but fail to regenerate, and eventually become atrophy. In the recent years, there has been a surge of interests to examine how neurons respond to injury and which factors are important for protecting neurons from damage and for promoting injured axons to regenerate. In this mini-review, I focus on the findings using the nematode *Caenorhabditis elegans* to discover molecular genetic pathways in synapse formation and axon regeneration.

## 1 *C. elegans* as a model organism

In a brief and informal letter, dated June 5th, 1963, to Max Perutz, the Director of the Laboratory of Molecular Biology at Cambridge, UK, Sydney Brenner wrote: “It seems to me that, both in development and in the nervous system, one of the serious problems is our inability to define unitary steps of any given process.”… “ The experimental approach that I would like to follow is to attempt to define the unitary steps…using the techniques of genetic analysis.”…”I would like to tame a small metazoan organism to study development directly” [3]. Brenner eventually chose *Caenorhabditis elegans* because it is amenable to genetics, its transparency is suited for light microscopy, its nervous system is small and also can be fixed to produce beautiful images using electron microscopy. Indeed, by painstakingly tracing individual neuronal processes from >8,000 serial ultra-thin sections and ingeniously interpreting anatomical contacts, John White deduced “The Mind of a Worm” [4], the first complete connectomics of an animal. Over the past four decades, genetic studies of *C. elegans* have led to the discovery of many fundamental principles in our understanding of the development and function of the nervous system.

## 2 Genetic dissection of synapse formation

The center of the synapse is generally referred to as the active zone. The presynaptic active zone holds the first importance for fast action in neurotransmission. They are morphologically defined by electron-dense specializations, and are composed of complex protein interaction network with a sole purpose to tether synaptic vesicles to the right location and maintain them to proper release state [5].

*C. elegans* displays sinusoidal locomotion. In the first screen for mutant *C. elegans,* Sydney Brenner isolated *uncoordinated* animals, and mapped them to over 70 genetic loci [6]. Early studies of many *unc* mutants revealed genes that primarily function in body muscle contractions. Molecular cloning of several *unc* loci initially defined novel protein families, which were later shown to be the key components of synaptic vesicle release machinery, such as the UNC-13/mUnc13 and UNC-18/mUnc18 proteins. Targeted identification of the genes that specifically function in synaptic transmission was greatly aided in subsequent screens for drug-resistant mutants; in particular, characterizations of *ric* mutants, for resistant to aldicarb, an inhibitor of acetylcholinesterase [7], uncovered many genes regulating pre-synaptic release. Nonetheless, majority of the *unc* and *ric* mutants do not alter synapse formation.

The discovery and development of green fluorescent protein, GFP, set the era to see things in a living cell [8]. To investigate how synapses are built, we first generated a simple marker by fusing GFP to the synaptic vesicle protein synaptobrevin. In live transgenic *C. elegans*, the chimera proteins appear as discrete fluorescent puncta, with each punctum representing a single presynaptic terminal [9]. We then performed forward genetic screening by visually inspecting the synaptic fluorescent puncta using fluorescent microscopy. The first set of Synapse defective, “*Syd*”, mutants affected synapse number and morphology. Following the identification of the genes and studies of their proteins, we found that the proteins encoded by the *Syd* genes are all localized to the presynaptic terminals. Moreover, these proteins reside in different subdomains and regulate different aspects of synapse formation. For example, SYD-2 is a member of the conserved Liprin-α (Lar RPTP interacting protein) proteins, and is localized to the center of presynaptic active zone. Changing *syd-2* activity using genetic loss- and gain- of function mutations alters the size and shape of the presynaptic active zone [10–12]. RPM-1 (Regulator of Presynaptic Morphology-1), together with its homologs Drosophila Highwire and human Pam, defines a novel conserved family, known as PHR proteins [13]. *rpm-1* mutants show reduced synapse number and altered synapse organization, as well as distinct axon patterning defects [14]. Studies on fly Highwire and mouse Phr reveal broad functions in the development and maintenance of the nervous system [13]. Subsequent work from other researchers using similar synaptic labeling methods for different types of *C. elegans* neurons further expanded the repertoire of genes regulating synapse specificity, synaptic trafficking, and synapse maintenance [15].

The awesome power of *C. elegans* genetics is not only demonstrated in discovering genes based on function, but also instrumental for dissecting signaling pathways. To understand how the *Syd* genes regulate synapse formation, we further performed genetic modifier screens, taking advantage of a distinct synthetic movement defect of *rpm-1; syd-2* double mutants [16]. Suppressor mutations can be readily isolated by movement improvement, and further examination on synapse morphology enabled us to sort a large number of mutations into specific groups. Such selection-based screens are commonly known as “screens from heaven” [17]. Interestingly, nearly all of the suppressor mutations of *rpm-1; syd-2* were specific to *rpm-1.* Together, they affected six genes that constitute a single MAP kinase cascade, consisted of the MAPKKK DLK-1, MAPKK MKK-4, MAPK PMK-3, MAPKAP2 MAK-2, a cofactor for PMK-3 and a bZip domain transcription factor CEBP-1 [16,18,25]. The PHR proteins are predicted to be E3 ligases [13]. Using biochemical and cell biological approaches, we demonstrated that DLK-1 is a physiological relevant substrate of RPM-1 [18]. The DLK-1 kinase cascade is conserved throughout evolution. Moreover, the ubiquitin-mediated regulation of DLK kinase by PHR E3 ligase is also evolutionarily conserved. The essence of this regulation is to maintain an optimal signaling output of the DLK-1 MAP kinase during synapse formation. Nonetheless, complete loss of function animals for the *dlk-1* pathway live as healthy animals, enabling our investigation for its roles in mature nervous system.

## 3 Axon regeneration in *C. elegans*

Understanding how nerves regenerate is an important step towards developing treatments for debilitating injuries, but investigation on axon regeneration has traditionally been carried out in large animals where mechanical injury can be made. *C. elegans* is microscopic, with the largest animal being 1mm long, and the diameter of axons is <1 μm. To impose injury to axons in living *C. elegans,* we established the fementosecond laser axotomy technology [19]. The basis of this technology is the high peak intensities of the laser pulses, which reduce the energy threshold for tissue ablation and enable precise surgery with a low-energy source. Following laser severing of single axon labeled by GFP, the injured axons initiate rapid response by forming filopodia and growth cones. Interestingly, the regrowth from injured axons is error-prone and misguided, resembling those in higher vertebrates, such as peripheral nerve regeneration. We further identified a rapid calcium transient induced by laser injury, and demonstrated that the calcium transient acts together with cAMP to promote regrowth and some degree of axonal fusion and synapse formation [20]. These effects of elevating cAMP levels share similarities to those in vertebrate axon regeneration, supporting the utility of the *C. elegans* injury model for mechanistic studies.

To identify genes and pathways that function in axon regeneration, we took advantage of the *C. elegans* genetic mutants and systematically examined the roles of genes conserved with human. About one third of *C. elegans* genes have human orthologs. We screened >1,000 genes for which null mutants show no discernable defects in the development of the nervous system. These genes fall into all categories of cellular function. We found about 15% of genes are required for regrowth [21], indicating that regeneration involves coordinated regulation of multiple pathways. Once again, the awesome power of *C. elegans* genetics has guided the mechanistic investigation of novel axon regeneration pathways.

Our recent studies on a gene named EFA-6 (for Exchange Factor for Arf6, due to the presence of the Sec7 homology domain) have revealed a previous unknown mechanism that regulates injury-induced microtubule (MT] dynamics [21,22]. Loss of *efa-6* results in increased axon regrowth, and overexpression of *efa-6* inhibits axon regrowth. In healthy neurons EFA-6 proteins reside to plasma membrane due to the presence of a Pleckstrin homology (PH) domain. Strikingly, upon injury to axons, EFA-6 undergoes a rapid re-localization, forming axonal puncta [22]. Such injury-triggered re-localization is correlated to the activity of EFA-6 in inhibiting axon regrowth. Based on the protein homology to Sec7, EFA-6 is predicted to have guanine exchange factor function for small GTPase Arf6. However, to our surprise, we found that the axon re-growth inhibitory activity of EFA-6 is independent of the Sec7 domain, and instead, resides in the N-terminal domain of EFA-6, which is predicted to be intrinsically disordered. In recent years, intrinsic disordered proteins have emerged as versatile signaling hubs to nucleate interacting proteins. Indeed, we found that EFA-6 binds two conserved MT binding proteins that belong to the families of Transforming Acidic Coiled-Coil proteins and the Doublecortin proteins, respectively. MTs are intrinsically polarized, with the plus end being dynamically growing and shrinking. Using a MT plus end marker to measure MT dynamics *in vivo*, we found that injured axons display increased MT dynamics in *efa-6(lf)* and decreased dynamics in *efa-6(gf)*. These studies thus uncover a mechanism by which axonal translocation of EFA-6 under traumatic injury activates its activity to regulate dynamic interactions between MT binding proteins to influence regenerative axon growth. Our findings are timely for axon regeneration studies in mammalian injury models. Several studies have shown that pharmacological interventions of MTs using taxol or epothilone can inhibit formation of glial scars and effectively stabilize injured axons, thereby promoting spinal cord axon regeneration [23,24]. In higher mammals including human, the EFA6 family expands to four genes, all of which appear to be widely expressed in nervous system. Understanding their function *in vivo* may lead to the development of new therapeutic targets.

## 4 Re-discovering DLK-1 kinase pathway in axon regeneration

MAP kinases play critical roles in nearly all biological processes. *C. elegans* share 153/212 subfamilies with human, providing close homologs for 81% of all human kinases. In the screen for axon regeneration factors, we unexpectedly found that the DLK-1 kinase cascade is essential for axon regrowth. Loss of *dlk-1* and its downstream components blocks growth cone formation from severed axons, whereas elevating the activity of *dlk-1* pathway accelerates growth formation and increases regrowth extent [25]. Moreover, DLK-1 promotes MT dynamics and growth induced by laser injury [26]. How might DLK-1 kinase be activated by traumatic injury? Through extensive genetic studies, we have identified a mechanism involving auto-inhibitory regulation of DLK-1. Two mRNA isoforms of *dlk-1* are produced through alternative polyadenylation, encoding a full length protein DLK-1L and a C-terminal truncated protein DLK-1S, respectively [27]. Interestingly, we found one class of genetic loss of function mutations of *dlk-1* affects only the C-terminal domain of DLK-1L, even though in these mutants DLK-1S are produced. Interestingly, DLK-1S and DLK-1L can bind each other, with the heteromeric interaction of DLK-1L and DLK-1S being stronger than homomeric binding of DLK-1L. Moreover, overexpression of DLK-1S inhibits axon regrowth, in part by inhibiting the activity of DLK-1L. Elevating calcium levels can reduce DLK-1L and DLK-1S heteromeric interaction, favoring the formation of DLK-1L homodimer, suggesting that activation of DLK-1L can be directly influenced by injury induced calcium influx. Intriguingly, the C-terminal domain of the DLK-1L contains a small domain that is conserved in human DLK family of kinases. In the past a few years, evidence emerges that DLK kinases play important roles in axon response to injury, in a manner depending on neuron type and injury paradigm [28]. It is of great interest to examine in-depth the many roles that these stress-sensing kinases play in building and protecting the nervous system.

## 5 Conclusion

The awesome power of *C. elegans* genetics has made discovery-based research immensely successful. Identification of genes provides the entry point into many areas of fascinating biology. Mechanistic investigation using this organism offers superb advantage at the resolution of single synapse, single axon, and single cell. The “hub” genes are used and re-used at different time under specific cellular contexts, nonetheless, the genetic logic remains the same. A major goal in the future is to link synaptogenesis in developing nervous system to repair circuit function under debilitating conditions. Moreover, as the increasing number of genetic variants reported in many human diseases is increasing rapidly, a challenge is how to determine the functional impact of such variants. Research using *C. elegans* will continue to reveal deep insights into the fundamental biology.
